# Prevalence of *Salmonella* serovars isolated from reptiles in Norwegian zoos

**DOI:** 10.1186/s13028-020-0502-0

**Published:** 2020-01-09

**Authors:** Ane Mohn Bjelland, Lena Maria Sandvik, Marianne Muri Skarstein, Linn Svendal, John James Debenham

**Affiliations:** 1 Section for Microbiology, Immunology and Parasitology, Department of Food Safety and Infection Biology, School of Veterinary Science, Norwegian University of Life Sciences, Oslo, Norway; 2 Department of Companion Animal Clinical Science, Faculty of Veterinary Science, Norwegian University of Life Sciences, Oslo, Norway

**Keywords:** Norway, Prevalence, Reptile, *Salmonella*, Zoonosis

## Abstract

**Background:**

Reptiles are known to be asymptomatic carriers of *Salmonella* spp. in their gastrointestinal mucosa and a variety of *Salmonella* serovars including exotic serovars mainly associated with reptiles as well as human pathogenic serovars have been isolated. There are many case reports of reptile-associated *Salmonella* infections worldwide, including one case in Norway in 2000. In August 2017, there was a legislative change in Norway that allowed more permissive reptile ownership and legalized the keeping of 19 different reptile species by private persons. There has been a concern that this new legislation will lead to an increase in reptile-associated salmonellosis in Norway, however knowledge is lacking on the occurrence of *Salmonella* spp. in Norwegian reptiles. The aim of this study was therefore to investigate the prevalence of *Salmonella* spp. in captive reptile species in Norway, identify the serovars and evaluate their zoonotic potential. Thus, cloacal swabs were taken from 53 snakes, 15 lizards and 35 chelonians from three Norwegian zoos, and assessed for the presence of *Salmonella* spp. by culture, biochemical testing and serotyping.

**Results:**

In total, 43% of the reptiles were shedding *Salmonella* spp., with a prevalence of 62%, 67% and 3% in snakes, lizards and chelonians, respectively. A total of 26 different serovars were found, including *Salmonella enterica* spp. *enterica* (40%) and *S. enterica* spp. *arizonae* (4%), both of which are considered to have a high zoonotic potential. *S. enterica* spp. *diarizonae, salamae* and *houtenae* were also identified, however these serovars are considered to have a lower zoonotic potential.

**Conclusions:**

The current study demonstrates that captive Norwegian reptiles are carriers of potentially zoonotic *Salmonella* spp. Given the increasing popularity of reptiles as pets and the legislative change, reptile-associated salmonellosis could become an increasingly important public health concern in Norway. Adequate public information about the risk of *Salmonella* infection as well as preventive measures to avoid *Salmonella* transmission from reptiles to humans is needed. The risk of *Salmonella* infection is considered low when recommended precautions are taken and good hygiene exhibited.

## Background

*Salmonella* is a Gram-negative bacterium of the Enterobacteriaceae family that can survive for weeks in dry environments and for months in water [1]. The bacterium is generally considered a normal constituent of the reptilian intestinal microbiota. Some studies report prevalence of *Salmonella* spp. in reptiles up to 90%, with a wide variety of serovars being identified [2–11]. These includes several serovars primarily associated with reptiles as well as non-host adapted serovars, including well‐known zoonoses such as *Salmonella* serovar Enteritidis and *S.* ser. Typhimurium [12–14]. Reptiles excrete *Salmonella* spp. in their feces intermittently and the bacterial load they shed is reported to increase during periods of stress e.g. transportation, handling, illness, high animal density and otherwise suboptimal environments [10].

According to the World Health Organization [15], *Salmonella* is one of the major global causes of diarrheal diseases, and usually associated with consumption of contaminated food products of animal origin. In the USA, about 1.35 million cases of illness, 26 500 hospitalizations, and 420 deaths occur every year due to nontyphoidal *Salmonella* infection [16], and direct contact with animals is estimated to account for 11% of *Salmonella* enteritis cases [17]. Salmonellosis in mammals causes a range of symptoms from diarrhea, vomiting, and fever, to life threatening septicemia [18]. Infection in humans is most severe in young children, the elderly and those with a reduced immune system [15]. Most of the *Salmonella* isolates that cause disease in mammals belong to *Salmonella enterica* spp*. enterica*. A few serovars of this subspecies are strictly human pathogens without an animal reservoir. The remaining *S. enterica* spp*. enterica* serovars are considered zoonotic or potentially zoonotic [14, 19]. The most common serovars infecting humans worldwide are *S.* ser*.* Typhimurium and *S.* ser. Enteritidis [15]. Reptiles infected by *Salmonella* spp. do not usually develop disease, however clinical salmonellosis occurs in reptiles and is generally provoked by an underlying primary cause of disease, although primary disease can occur [20].

Exotic pet ownership has become increasingly popular worldwide [21, 22]. The European Union member states are among the largest importers of reptiles, and in the USA, 4.7 million households own a reptile [23, 24]. Simultaneously, reptiles and amphibians are estimated to account for 6% of all *Salmonella* infections in the USA and Europe and may be increasing [13, 25–28]. During 2006–2014, a total of 15 multistate outbreaks of turtle-associated salmonellosis in humans were reported in the USA [28]. Reptiles kept as pets are also potential sources of *Salmonella* infection for other companion animals, such as dogs and cats, which can contribute to the spread of this pathogen in the environment and increase the risk of infection for humans [29].

In Norway, the occurrence of human salmonellosis is low compared to other countries with about 1000 reported cases annually [30]. In August 2017, pet ownership of 19 different reptile species including snakes (nine species), lizards (seven species) and chelonians (three species) was legalized in Norway. Prior to this, permission to hold reptiles was given almost exclusively to zoos and aquaria, with privately owned reptiles being prohibited. Nevertheless, illegal hold of reptiles in private homes existed and a single case of reptile-associated salmonellosis had been reported in Norway [31]. There has been a concern that this new legislation will lead to increased occurrence of salmonellosis in Norway. The risk of reptile-associated salmonellosis in humans depends on several factors such as *Salmonella* spp. prevalence, serovar predominance and pathogenicity, as well as exposure and immunocompetence of the human [11, 13]. However, little is known about the prevalence and serovar predominance in Norwegian reptiles. Therefore, the aim of this study was to investigate the prevalence of *Salmonella* spp. in captive reptile species in Norway, identify the serovars isolated from this population, and evaluate their zoonotic potential.

## Methods

### Animal selection

During 2016, reptiles were sampled from three zoos (referred to as A, B and C) in Norway and examined for *Salmonella*. Exclusion criteria comprised animals (a) with a cloaca too small for swab insertion, (b) showing signs of disease (c) treated with antibiotics within the last 30 days, and (d) that shared cage with reptile(s) treated with antibiotics the last 30 days. Also, snakes that (e) showed signs of ecdysis, and (f) were fed the same day as sampling, were excluded from the study. All other reptiles in the three zoos were selected for sampling and included 35 chelonians, 15 lizards and 53 snakes representing 22 different species. The classification and numbers of reptiles sampled from each zoo are described in Table 1. All animals were considered healthy at the time of sampling based on daily observations by the zookeepers over the previous month, and physical examination by a veterinarian at time of sampling.

**Table 1:** Species and zoo location of reptiles sampled in the study to investigate the prevalence of *Salmonella* spp. in captive reptile species in Norway

Suborder	Family	Species		No. of reptiles			
				Zoo A	Zoo B	Zoo C	Total
Cryptodira	Testudinidae	Leopard tortoise	*Stigmochelys pardalis*	2			2
		Hermann’s tortoise	*Testudo hermanni*	1	2		3
		Russian tortoise	*Testudo horsfieldii*	3			3
	Emydidae	Red-eared slider	*Trachemys scripta elegans*	16		4	20
		Yellow-bellied slider	*Trachemys scripta scripta*	5		2	7
			No. of chelonians	27	2	6	35
Iguania	Iguanidae	Green iguana	*Iguana iguana*	2	1		3
	Agamidae	Central bearded dragon	*Pogona viticeps*		2	2	4
	Chamaeleonidae	Panther chameleon	*Furcifer pardalis*			1	1
Scincomorpha	Lacertidae	Ocellated lizard	*Timon lepidus*			3	3
	Scincidae	Blue-tongue skink	*Tiliqua nigrolutea*		1	3	4
			No. of lizards	2	4	9	15
Serpentes	Boidae	Common boa	*Boa constrictor*	2	3	1	6
		Cuban boa	*Chilabothrus angulifer*		1		1
		Rainbow boa	*Epicrates cenchria*		1		1
	Colubridae	Corn snake	*Pantherophis guttatus*	2	2		4
		California kingsnake	*Lampropeltis getula californiae*	1	1	1	3
		Hognose	*Heterodon nasicus*		2	3	5
	Pythonidae	Royal python	*Python regius*	1	8	8	17
		Carpet python	*Morelia spilota*	1	2	3	6
		Indian python	*Python molurus*	1	3	1	5
		Angolan python	*Python anchietae*		2		2
		Green tree python	*Morelia viridis*			1	1
		Spotted python	*Antaresia maculosa*		2		2
			No. of snakes	8	27	18	53
			No. of reptiles from each zoo	37	33	33	103

### Sample collection and processing

Depending on animal size, regular or minitip bacteriology swabs of soft rayon were used for fecal sampling (Copan Diagnostics Inc., Murrieta, CA, USA). The live animals were physically restrained, and a swab was inserted into the cloaca and gently rotated longitudinally. Swabs were then placed into Amies agar gel medium with or without charcoal (Copan Diagnostics Inc.), stored at 4 °C and processed within 24 h.

All growth media and biochemical tests used were produced in-house at the Department of Food Safety and Infection Biology, Norwegian University of Life Sciences (Oslo, Norway). For recovery of *Salmonella* spp. cloacal swabs were direct-plated on selective Bromthymolblue-Lactose-Agar (BTBL) (Brolac, Cat.-No. 1639, Merck, Darmstadt, Germany) and incubated at 35 °C for 24 h. For enrichment of *Salmonella* spp., the swabs were first placed into 4 mL buffered peptone water (BPW, Merck) and cultivated at 35 °C for 24 h before 1 mL inoculum was transferred to 4 mL selenite broth (Difco™ Selenite Cystine Broth, BD Diagnostics, Sparks, MD, USA) and further incubated at 42 °C [32, 33]. Every day for three days, a sterile plastic bacterial loop was used to transfer 1 µL aliquot of enriched broth to a BTBL plate followed by incubation at 35 °C for 24 h. Oxidase-negative (Oxidase Strips, Oxoid, Cambridge, UK) and non-lactose-fermenting bacteria (blue), and thus suspected *Salmonella* colonies, were streaked onto urea agar (Oxoid) and triple sugar iron (TSI) agar (Difco, BD Diagnostics) and incubated at 35 °C for 24 h. Samples were considered positive for *Salmonella* based on a negative urea result and the production of hydrogen sulfide in the TSI test. Suspect colonies were also analyzed using the API20E kit after the manufacturer’s description (BioMérieux, Marcy-l’Étoile, France). One single colony were isolated from each BTBL plate for further identification when *Salmonella* was suspected. Presumptive colonies of *Salmonella* were restreaked onto blood agar plates (blood agar base no. 2 (Oxoid) supplemented with 5% bovine blood) and submitted to the Norwegian Veterinary Institute where the isolates were serotyped by agglutination tests with antisera (SIFIN, Berlin, Germany and Statens Serum Institut (SSI), Hillerød, Denmark) according to the White-Kauffmann scheme [34]. *Salmonella* subspecies I (*S. enterica* ssp*. enterica*) were identified as named serovars, except one sample.

### Evaluation of zoonotic potential

A literature review was conducted by using publications indexed at PubMed as well as other Internet resources to evaluate the zoonotic potential of the *Salmonella* serovars isolated in the present study. Database searches were conducted from February 2016 to July 2019 with the search terms “reptile”, “zoonosis”, “Salmonella”, in addition to the name of specific serovars. *Salmonella* serovars that have been reported to cause illness in otherwise healthy adults with normal immune status were considered to have a high zoonotic potential. Serovars reported to cause disease in the immunonaieve or immunocompromised individuals were considered to be of moderate zoonotic potential, and those serovars that only have been reported to cause disease in a few individuals were considered to have a low zoonotic potential.

### Statistical analysis

Confidence intervals (CI) for binominal distribution were calculated using online software available at Statpages.net [35]. A two-tailed P-value was calculated from a 2 × 2 contigency table by Fisher’s exact test using the GraphPad QuickCalcs software for statistical comparisons between groups for the prevalence of *Salmonella* [36]*.* A P-value ≤ 0.05 was considered statistically significant.

## Results

### Prevalence of *Salmonella* spp. from cloacal samples

A total of 44 out of 103 cloacal samples (43%, CI 33–53%) were *Salmonella*-positive, as determined by biochemical tests and serotyping. *Salmonella* spp. were isolated from 16 of the 22 different reptile species (73%) included in this study.

In snakes and lizards, 62% (CI 48–75%) and 67% (CI 38–88%) of samples were positive for *Salmonella*, respectively. In chelonians, *Salmonella* sp. was only identified in one sample (CI 0–0.15%) originating from a Hermann’s tortoise (*Testudo hermanni*). The prevalence of *Salmonella* was significantly lower in chelonians than in lizards and snakes (P < 0.001), and this difference remained even if the results of a large group of *Trachemys scripta* (n = 21) that were housed together were excluded.

*Salmonella* spp. were identified in 24% of the tested reptiles in Zoo A (CI 12–41%), 52% in Zoo B (CI 34–69%) and 55% in Zoo C (CI 36–72%). In Zoo A, all snakes and one lizard (50%) were *Salmonella*-positive. All 27 chelonians that were tested in the same zoo were found to be negative. In Zoo B, *Salmonella* spp. was identified in 37% of the snakes, 75% of the lizards and in 50% of the chelonians. In Zoo C, 67% of both the snakes and the lizards were *Salmonella*-positive, whilst all chelonians were negative.

### Serotyping of *Salmonella* isolates

Of the samples that tested positive for *Salmonella*, a single isolate was identified from each sample except one, originating from a Royal python (*Python regius*), where one isolate was identified by direct-plating and another isolate after enrichment. Thus, 45 isolates of *Salmonella* spp. were identified from the 44 positive cloacal samples. In total, 26 different serovars were identified by serotyping. *S. enterica* spp*. enterica* was the most frequently identified subspecies, comprising 40% of positive samples, followed by *S. enterica* spp*. diarizonae*, 36%, *S. enterica* spp*. salamae* 11%, *S. enterica* spp*. arizonae*, 4%, and *S. enterica* spp*. houtenae*, 2%. Three *Salmonella* serovars (7%) were of unknown subspecies (Table 2). The number of serovars identified from each subspecies is listed in Table 2.

**Table 2:** *Salmonella* species isolated from Norwegian zoo reptiles, serovars and their host, including numbers of each serovar and numbers of each host species

Species	Subspecies	Serovar	No. of isolates	Host		No. of hosts
*Salmonella enterica*	*enterica*	Kottbus	1	Hermann’s tortoise	*Testudo hermanni*	1
		Hadar	1	Central bearded dragon	*Pogona viticeps*	1
		Poano	1	Cuban boa	*Chilabothrus angulifer*	1
		Muenchen	5	Spotted python	*Antaresia maculosa*	2
				Carpet python	*Morelia spilota*	1
				Common boa	*Boa constrictor*	1
				Corn snake	*Pantherophis guttatus*	1
		Redlands	1	Corn snake	*Pantherophis guttatus*	1
		9,12:eh:-	1	Blue-tongue skink	*Tiliqua nigrolutea*	1
		Cotham	1	Central bearded dragon	*Pogona viticeps*	1
		Lome	1	Royal python	*Python regius*	1
		Paratyphi B var Java	6	Royal python	*Python regius*	4^a^
				Green tree python	*Morelia viridis*	1
				Carpet python	*Morelia spilota*	1
	*arizonae*	44:z4, z23:-	1	Corn snake	*Pantherophis guttatus*	1
		51:z4z23:-	1	Carpet python	*Morelia spilota*	1
	*diarizonae*	57:c:z	2	Royal python	*Python regius*	1
				Indian python	*Python molurus*	1
		47:k:z35	1	Blue-tongue skink	*Tiliqua nigrolutea*	1
		48:z52:enz15	1	Carpet python	*Morelia spilota*	1
		48:1, v:1,5	1	Common boa	*Boa constrictor*	1
		48:1w:1,5,7	1	Common boa	*Boa constrictor*	1
		48:c:z	1	Green iguana	*Iguana iguana*	1
		50:r:z	1	Royal python	*Python regius*	1
		65:k:z53	7	Royal python	*Python regius*	1
				Hognose	*Heterodon*	3
				Carpet python	*Morelia spilota*	2
				Central bearded dragon	*Pogona viticeps*	1
		14:z10:z	1	California kingsnake	*Lampropeltis getula californiae*	1
	*salamae*	18:z4,z23:-	4	Royal python	*Python regius*	2^a^
				Ocellated lizard	*Timon lepidus*	1
				Central bearded dragon	*Pogona viticeps*	1
		16:gm t:-	1	Angolan python	*Python anchietae*	1
	*houtenae*	44:z4z23:-	1	Ocellated lizard	*Timon lepidus*	1
*Salmonella* sp.	Unknown	49:1w:1,5,7	1	Indian python	*Python molurus*	1
		28:z52:z53	1	Blue-tongue skink	*Tiliqua nigrolutea*	1
		51:i:1,2	1	Royal python	*Python regius*	1

^a^Two different *Salmonella* isolates (*S*. ser. Paratyphi B var Java and *S*. *enterica* ssp*. salamae* ser. 18:z4,z23:-) were identified originating from a common Royal python (*Python regius*) host. Thus, 45 isolates of *Salmonella* spp. were identified from 44 positive reptile hosts

Whilst several of the *Salmonella*-positive reptiles shared their cage with other animals of the same species, cohabiting reptiles carrying the same serovars of *Salmonella* were only identified in 2 of 14 cages in this study, both cages holding snakes. In seven (50%) of the cages, both *Salmonella*-positive and *Salmonella*-negative animals were identified.

### Zoonotic potential of identified *Salmonella* serovars

*Salmonella* serovars identified in this study and their zoonotic potential are listed in Table 3. Sixteen reptiles (15.5%) carrying *Salmonella* serovars with a high zoonotic potential were identified. These serovars were; *S*. ser. Paratyphi B var Java, *S*. ser. Muenchen, *S*. ser. Cotham, *S*. ser. Kottbus, *S*. ser. Hadar, *S. enterica* spp*. arizonae* 44:z4, z23:- and *S. enterica* spp*. arizonae* 51:z4z23:*.* Serovars of *S. enterica* spp*. diarizonae* and *S. enterica* spp*. houtenae* that were considered to have a moderate zoonotic potential were isolated from 17 reptiles (16%).

**Table 3:** *Salmonella* subspecies and serovars identified in this study and their zoonotic potential

Species	Subspecies	Serovar	Zoonotic potential	No	References
*Salmonella enterica*	*enterica*	Paratyphi B var Java	High	6	[39–41]
		Muenchen		5	[42, 55]
		Cotham		1	[43, 44]
		Kottbus		1	[56]
		Hadar		1	[48–50, 55]
		Lome	Low	1	[57]
		Poano		1	[58]
		Redlands		1	N/A
		9,12:eh:-	Unkown	1	N/A
	*arizonae*		High	2	[45]
	*diarizonae*		Moderate	16	[21, 53, 54]
	*houtenae*		Moderate	1	[51, 52]
	*salamae*		Low	5	[59, 60]
*Salmonella* sp.	Unknown		Unknown	3	N/A

*N/A* not applicable

## Discussion

### Prevalence of *Salmonella* spp. in captive Norwegian reptiles compared to other countries

The overall prevalence of *Salmonella* in captive Norwegian reptiles (43%, CI 33–53%) is consistent with the spectrum of prevalence’s reported globally: Japan (74%) [6], Germany/Austria (54%) [3], Italy (51 and 57%) [4, 22], Australia (47%) [10], Denmark (35%) [11], Taiwan (31%) [8], Trinidad (31%) [2], Republic of Korea (30%) [7], Croatia (13%) [37] and New Zealand (11%) [9]. The variation in reported *Salmonella* prevalence amongst different reptile populations may represent a true different in infection status, for instance Scheelings et al. [10] found a higher prevalence of *Salmonella* in reptiles held in captivity (47%) compared to wild reptiles (14%), although this is yet to be confirmed by other studies. Unfortunately, whilst one can speculate about factors that may influence the true infection status, such as wild vs captive, season, climate, environment, other diseases and diet, little evidence is available on how these factors truly affects *Salmonella* infection. Further limiting the usefulness of comparing results between studies is the considerable variation in experimental design and the use of different diagnostic techniques. For instance, whilst we used cloacal swabs, other studies have used fecal samples, oral swabs and skin swabs [3, 4, 6, 37]. Additionally, the phenomenon of intermittent shedding probably accounts significantly for the variability in detection rates between authors [12]. In the studies from Croatia, New Zealand and Italy, sampling was performed by the animal’s owner, which could have given some more unreliable results and thus lower prevalence [4, 9, 22, 37].

In general, *Salmonella* prevalence is reported to be higher in snakes than in lizards or chelonias [3, 6, 8, 10, 11]. In this study, no significant difference in *Salmonella* prevalence was found between snakes and lizards, however this may have been due to inadequate sample size. In contrast, *Salmonella* prevalence in chelonians was significant lower compared to the two other groups, which is consistent with results from Germany/Austria, Australia, New Zealand and Taiwan [3, 8–10]. However, reported *Salmonella* prevalence in chelonians varies highly between studies (3–72%) [3, 6, 8–11, 22, 37]. The prevalence within Chelonia in this study may have been skewed by a very large group of *Salmonella*-negative *Trachemys scripta* that were housed together and constituted over half the total number Chelonia included in the study.

The occurrence of *Salmonella* spp. in Norwegian production and companion animals, as well as animal feeds and products is very low compared to most other countries [30]. This favorable situation does not however include the captive Norwegian reptiles. This study shows that the *Salmonella* prevalence in Norwegian reptiles is similar to the prevalence reported in other countries. Most captive reptiles in Norway are imported from other countries and might have been exposed to *Salmonella* spp. early in life when the intestinal microbiota is established, thus becoming permanent carriers of the bacteria. Only a few wild-living reptile species exist in Norway, however screening of these animals would be of great interest to further elucidate the relationship between *Salmonella* spp. and reptiles.

This study represents the first investigation into the prevalence of *Salmonella* in Norwegian reptiles. Ideally, investigation of the risk for reptile associated salmonellosis would be based upon a population of pet reptiles, not zoological collections. However, at the time of sampling, hold of reptiles in private households was illegal in Norway, thus making it complicated to access this population. Instead, reptiles kept in different zoos in Norway were studied to evaluate the risk of zoonotic transmission of *Salmonella* spp. to visitors and employees. Reptiles kept in Norwegian zoos often originate from private homes and end up being relocated to a zoo after confiscation by the Norwegian Food Safety Authority. As such, these results can serve as a proxy for *Salmonella* in reptiles in private homes. However, little is known about how the intestinal microbiota is influenced by housing conditions and other environmental factors. Also, although all zoos in this study invited their visitors, including children, to hold and/or touch the reptiles the interaction with animals is probably more intense in private holdings and precautions less than in zoos. Thus, the zoonotic risk of salmonellosis may be higher in private homes compared to zoos.

### *Salmonella* serovars in Norwegian reptiles

In total, 45 *Salmonella* isolates were identified in 44 different individuals. Out of these, 18 (40%) and 16 (36%) isolates were of subspecies *enterica* and *diarizonae*, respectively, which is consistent with other studies [3, 6, 9–11]. *S. bongori* and *S. enterica* spp*. indica.* were not identified in this study, similar to previous reports [3, 6, 9, 10, 37]. The results documented in the present work as well as previous studies indicates that a great diversity of different *S. enterica* subspecies and serovars infect reptiles. Routinely, only one single colony was isolated from each sample, thus this investigation was not designed to detect a diversity of *Salmonella* subspecies and serovars in each single reptile’s intestinal microbiota. Nevertheless, two different subspecies were identified from the same animal on one occasion. A diversity of *Salmonella* subspecies and serovars in the reptilian intestine is previously described, and although a single serovar has been the most frequent finding, up to four different serovars have been reported from the same animal [3, 6].

This work does not clarify if *Salmonella* serovars transmit between the individual reptiles. Identical serovars of *Salmonella* in cohabiting reptiles were only identified in 2 of 14 cages in this study, however the study design does not exclude the possibility for unidentified *Salmonella* spp. in both the *Salmonella*-positive as well as the *Salmonella*-negative reptiles. In half of the cages, both *Salmonella*-positive and *Salmonella*-negative animals were identified. The fact that *Salmonella* excretion is intermittent represent a potential source of false negatives in prevalence studies, particularly if only one sample is taken [29]. Thus, a *Salmonella*-free status may have been a misinterpretation. By testing each individual multiple times, higher prevalence and diversity of *Salmonella* spp. could have been detected.

### Zoonotic potential of identified *Salmonella* serovars

*Salmonella* is one of the most common and important zoonoses in the world. However, the pathogenicity and zoonotic potential of *Salmonella* varies between different subspecies, serovars and strains [38]. In the current work, no pathogenicity studies on the different *Salmonella* isolates were performed and the evaluations of zoonotic potential should therefore be regarded with caution. *S. enterica* spp. *enterica* and *S. enterica* spp. *arizonae* were the subspecies with the highest zoonotic potential found in this study. *S. enterica* spp. *enterica* causes 99% of all human *Salmonella* infections [1], however none of the most common serovars identified to cause human salmonellosis in Norway (*S*. Enteritidis, *S*. Typhimurium, *S*. ser. Stanley, *S*. ser. Newport and *S*. ser. Java) were isolated in this study [30]. Nevertheless, *Salmonella* serovars with a high zoonotic potential were identified in 15.5% of the reptiles (Table 3). *S.* Paratyphi B var Java [39–41], *S*. ser. Muenchen [42], *S*. ser. Cotham [43, 44] and *S. enterica* spp. *arizonae* [45] are reported to cause several incidences of reptile-associated salmonellosis in otherwise healthy humans with normal immune status. *Salmonella* Kottbus and *S*. Hadar are serovars often related to human cases of food poisoning and have not been identified with reptile-associated salmonellosis [46–50]. However, close contact with reptiles carrying these *Salmonella* serovars could probably increase the risk of salmonellosis.

*Salmonella enterica* spp. *diarizonae* is found in high prevalence in both wild and captive reptiles and is frequently identified to be the cause of reptile-associated salmonellosis [21]. This subspecies, as well as *S. enteri*ca spp. *houtenae* were identified in 16% of the reptiles and considered to have a moderate zoonotic potential as most human cases occur in immunosuppressed individuals or children (Table 3) [21, 51–54]. In total almost 1/3 of the reptiles were identified as carriers of highly or moderately zoonotic *Salmonella* serovars. These results underline that all reptiles should be considered to be potential sources of zoonotic *Salmonella* spp.

## Conclusions

The current study demonstrates that captive Norwegian reptiles are carriers of potentially zoonotic *Salmonella* spp. Given the increasing popularity of reptiles as pets, reptile-associated salmonellosis could become an increasingly important public health concern in Norway. Adequate public information about the risk of *Salmonella* infection as well as preventive measures to avoid *Salmonella* transmission from reptiles to humans is needed.

## Data Availability

The datasets used and/or analysed during the current study are available from the corresponding author on reasonable request.

## Abbreviations

BTBL: bromthymolblue-lactose-agar
BPW: buffered peptone water
CDC: centers for Disease Control and Prevention
TSI: triple sugar iron
WHO: world Health Organization
